# Investigating the antimicrobial and antibiofilm properties of marine halophilic *Bacillus* species against ESKAPE pathogens

**DOI:** 10.1111/1758-2229.70027

**Published:** 2024-10-24

**Authors:** Monica M. Murphy, Eamonn P. Culligan, Craig P. Murphy

**Affiliations:** ^1^ Department of Biological Sciences Munster Technological University Cork Ireland

## Abstract

Antimicrobial resistance (AMR), known as the “silent pandemic,” is exacerbated by pathogenic bacteria's ability to form biofilms. Marine compounds hold promise for novel antibacterial drug discovery. Two isolates from preliminary saltwater environment screening demonstrated antimicrobial activity and were subsequently identified as *Bacillus subtilis* MTUA2 and *Bacillus velezensis* MTUC2. Minimum inhibitory concentrations (MICs), minimum biofilm inhibition concentrations (MBICs) and minimum biofilm eradication concentrations (MBECs) required to prevent and/or disrupt bacterial growth and biofilm formation were established for MRSA, *Staphylococcus aureus*, *Acinetobacter baumannii* and *Escherichia coli*. The metabolic activity within biofilms was determined by the 2,3,5‐triphenyltetrazolium chloride assay. Both *Bacillus* species exhibited unique antimicrobial effects, reducing MRSA and *S. aureus* planktonic cell growth by 50% and sessile cell growth for *S. aureus* and *E. coli* by 50% and 90%, respectively. No effect was observed against *A. baumannii*. Significant MBIC and MBEC values were achieved, with 99% inhibition and 90% reduction in MRSA and *S. aureus* biofilms. Additionally, 90% and 50% inhibition was observed in *E. coli* and *A. baumannii* biofilms, respectively, with a 50% reduction in *E. coli* biofilm. These findings suggest that the mode of action employed by *B. subtilis* MTUA2 and *B. velezensis* MTUC2 metabolites should be further characterized and could be beneficial if used independently or in combination with other treatments.

## Graphical abstract

Biofilms are responsible for approximately 80% of chronic infections, providing bacteria with an innate tolerance to antibiotics. However, once biofilm cells disperse, they quickly regain susceptibility. This study investigated the potential of bacterial isolates from aquatic environments to combat biofilm‐forming pathogens. These isolates not only inhibited biofilm formation but also demonstrated the ability to disrupt preformed biofilms of various clinically relevant pathogens, including MRSA, clinical *Staphylococcus aureus*, *Acinetobacter baumannii* and *Escherichia coli* strains.

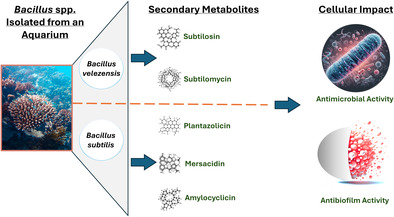

## INTRODUCTION

As bacterial pathogens continuously evolve, develop antibiotic resistance and drive the transfer of resistance genes, it is vital that new antimicrobials with alternative mechanisms action are found. For instance, the ESKAPE pathogens (*Enterococcus faecium*, *Staphylococcus aureus*, *Klebsiella pneumoniae*, *Acinetobacter baumannii*, *Pseudomonas aeruginosa* and *Enterococcus* spp.) are recognized for their high resistance to antibiotics and are associated with severe infections that carry a high risk of mortality (Tacconelli et al., 2018). These pathogens are listed as a priority by the World Health Organisation (WHO) due to the threat they pose to human health (Idris & Nadzir, 2023; Talebi Bezmin Abadi et al., 2019). Nosocomial ESKAPE pathogens employ a wide range of antimicrobial resistance mechanisms including the ability to form biofilms (Santajit & Indrawattana, 2016). These pathogens are responsible for a broad spectrum of chronic and persistent biofilm‐associated infections, thereby reducing the efficacy of conventional antibiotic treatments (De Oliveira et al., 2020).

Biofilms are classified as a homogenous or heterogeneous bacterial community encased in a protective polysaccharide matrix that rapidly colonize biotic and abiotic surfaces, conferring a critical advantage against antibiotic treatment (Wingender & Flemming, 2011). While planktonic cells are inherently more susceptible to antibiotic treatment, cells living in biofilms appear to have alternative mechanisms of bacterial antimicrobial resistance, referred to as biofilm antibiotic tolerance (BAT) (Jiang et al., 2020; Verderosa et al., 2019). However, several studies have shown that once the biofilm is disrupted, the dispersed cells regain their original antibiotic susceptibility (Kostakioti et al., 2013). Therefore, it is essential to develop innovative strategies that combat and disrupt biofilm formation in conjunction with antimicrobial compounds. Previously it has been elucidated that biofilms can promote persistence of human pathogens and are responsible for between 60 and 80% of microbial infections (Jamal et al., 2018).

Traditionally, isolation of novel antimicrobials was focused on soil microorganisms (Pérez et al., 2016). However, in recent years, the marine biosphere has gained considerable attention with marine microorganisms deemed important producers of biologically active peptides (Alvarez‐Yela et al., 2019; Stincone & Brandelli, 2020). Marine organisms exist in vastly different environments and must tolerate a range of environmental stressors, such as pressure, salinity and temperature, resulting in organisms that are structurally diverse and capable of producing novel chemical compounds, compared to microorganisms found in the terrestrial environment (Alvarez‐Yela et al., 2019; Choudhary et al., 2017). For instance, alkaloids, including the derivatives of polyoxygenated dihydropyrano[2,3‐c]pyrrole‐4,5‐dione such as pyranonigrin A and F isolated from the cultures of *Penicillium brocae* derived from a marine mangrove endophytic fungus (Meng et al., 2015), demonstrate promising antimicrobial properties against *S. aureus* and aqua‐bacteria *V. harveyi* and *V. parahaemolyticus*. Terpenoids, like napyradiomycin B2–B4, isolated from marine‐derived *Streptomyces* strains (Cheng et al., 2013) also exhibit potential as alternative antimicrobial and antibiofilm treatments. Additionally, peptides, such as halistanol sulfate A and rodriguesines A and B isolated from the ascidian *Didemnum* spp. (de Lima et al., 2014), have demonstrated efficacy in antibacterial activity and biofilm inhibition against the caries etiologic agent Streptococcus mutants in vitro.

*Bacillus* species are ubiquitous in marine ecosystems, inhabiting many different ecological niches (Phelan et al., 2013; Stincone & Brandelli, 2020). In addition to producing a wide range of bioactive compounds, *Bacillus* species develop highly resistant endospores when exposed to extreme conditions, such as heat, desiccation, radiation and UV light (Martins et al., 2002; Singh et al., 2015; Tareq & Shin, 2017). Marine *Bacillus* species can produce a range of chemically diverse bioactive compounds, such as bacteriocins, described as antibacterial peptides that possess killing or inhibitory action against the growth of closely related bacteria (Silva et al., 2018) and polyketides, shown to possess considerable antimicrobial activity against human pathogens (Chakraborty et al., 2018). Secondary metabolites produced by *Bacillus* species have been shown to have different mechanisms of action which target the cell wall, plasma membrane and intracellular processes, or which interfere with cell adherence and quorum signalling (QS), a mechanism crucial for regulating cell‐to‐cell communication and co‐operation in biofilms (Tran et al., 2022). For example, in a study conducted by Dong et al. (Dong et al., 2002) it was demonstrated that *B. thuringiensis*, *B. mycoides* and *B. cereus* strains isolated form soil and plant samples deactivated the QS signalling molecule, autoinducer acyl‐homoserine lactone (AHL). Additionally, subtilosin, the cyclic lantibiotic protein produced by *B. subtilis* KATMIRA1033, can disrupt quorum sensing in Gram‐positive bacteria by reducing the level of autoinducer‐2 (AI‐2) (Algburi et al., 2017).

The present study aimed to screen, isolate and identify bacterial isolates from saltwater aquariums and to investigate the potential antimicrobial, antibiofilm and biofilm eradication effects of the cell‐free supernatant (CFS) against bacterial pathogens which are implicated in a wide range of hospital acquired infections.

## EXPERIMENTAL PROCEDURES

### Sample collection

Saltwater (SW) samples were collected in sterile jars from a SW aquarium. The aquarium housed a collection of large polyp stony (LPS) corals, including *Euphylia and Acanthastrea*, as well as soft corals such as *Zoanthids*, *Ricordea*, *Rhodactis*, *Gorgonian* and *Sarcophyton* (leather toadstools). The aquarium featured a specific salt content of 1.025 (equivalent to 35 parts per thousand), was maintained at a constant temperature of 27°C and illuminated by LED light (A1 Hydra 26 HD light). All samples were kept at room temperature and transported directly to the laboratory for analysis.

### Bacterial strains, reagents and growth conditions

Bacterial test strains were obtained from Munster Technological University (MTU) culture collection: methicillin‐resistant *Staphylococcus aureus* (MRSA) and four clinical *Staphylococcus* strains [MTU48, MTU100, MTU300 and MTUCC], *Acinetobacter baumannii* Ab003, *Acinetobacter baumannii* Ab0013 and *Escherichia coli* K‐12. Bacterial strains were grown in Tryptone Soy Broth (TSB Millipore, Batch No. 150461/352) supplemented with 0.5% glucose (TSBg; Sigma Aldrich, Lot #SZBF0820V). In all experiments, the effectiveness of the SW isolates was compared against ciprofloxacin (20 mg/mL; Sigma Aldrich), a known inhibitor of all bacterial strains in this study. Bacterial strains grown in sterile TSBg served as a negative control. All experiments were conducted in triplicate. Stocks of all bacterial strains were stored in 20% (v/v) glycerol at −80°C until experimental use.

### Isolation of microorganisms from salt water and subsequent deferred antagonism assay

To isolate microorganisms, each collected sample was serial diluted in sterile Dh_2_O and subsequently plated on to Tryptic Soy Agar (TSA) supplemented with 0.5% glucose (TSAg). Plating was performed in triplicate with plates incubated for 24 h at three separate temperatures: 20, 30 and 37°C. Following incubation, antimicrobial activity was examined by a deferred antagonism assay (Twomey et al., 2021). This was achieved by spotting distinct colonies from SW growth plates onto TSAg agar plates, and the plates were incubated overnight at 37°C. After 24 h, the plates were exposed to UV light for 30 min. Each plate was overlaid with sloppy agar (0.75%) seeded with 100 mL of 0.5 McFarland standards for each test strain under investigation. The plates were left to set, and then incubated upright at 37°C for 24 h. Zones of inhibition around colonies were taken as an indication of antimicrobial activity. Two isolates were identified as potential producer strains and were transferred to separate TSAg plates to confirm antimicrobial activity via a second deferred antagonism assay.

To prepare the cell free supernatant (CFS), 10 mL of broth was inoculated with one colony of each producer strain and incubated at 37°C for 24 h. Following overnight incubation, the cells were pelleted by centrifugation (4000×*g*, for 20 min at 4°C) and the supernatant was filter‐sterilized through a 0.2 μm pore size syringe filter (Millipore, Burlington, MA). CFS was stored at 4°C prior to use.

### Whole genome sequencing, assembly and genome annotation

Total DNA was isolated from an overnight culture of each of the two producer strains using TRIzol reagent (Thermo Fisher, Waltham, MA) according to the manufacturer's protocol. Whole genome sequencing (WGS), assembly and annotation were performed by MicrobesNG (Birmingham, UK) on the Illumina HiSeq platform. Reads were adapter trimmed using Trimmomatic v. 0.30 with a sliding window quality cut‐off of Q15 (Bolger et al., 2014). Paired reads were aligned to the annotated reference genome sequence using the Burrow‐Wheeler Aligner (bwa‐mem) in Samtools (Li, 2023) and BedTools (Ar & Im, 2010). The sequence data were deposited in NCBI GenBank. The sequence data were deposited in NCBI GenBank under accession numbers; *Bacillus subtilis* strain MTUA2 (JBBKZW000000000) and *Bacillus velezensis* MTUC2 (JBBKZX000000000). Average nucleotide identity (ANI) was used to determine closely related reference strains. This was achieved using the 16S rRNA gene of both isolates which were blasted on NCBI BLASTn using a threshold *E*‐value of ≤1^e‐6^ and a sequence identity of ≥70% (Choudhuri, 2014). Five of the closest‐related species of each isolate with their respective accession numbers (Table 1) were downloaded from the NCBI GenBank database and used for pairwise analysis. Subsequently, the genomes were annotated using RAST (https://rast.nmpdr.org/) (Aziz et al., 2008) and visualized using Artemis (Carver et al., 2012). Secondary metabolite biosynthetic gene clusters (BGC) were identified using BAGEL 4 (van Heel et al., 2018) and antiSMASH 7.0 (Antibiotics & Secondary Metabolite Analysis Shell) (Blin et al., 2023).

**TABLE 1:** Reference strains from NCBI analysed in this study for genome analysis.

Microbial strain	NCBI reference sequence
*Bacillus subtilis* strain H1	CP026662.1
*Bacillus subtilis* strain SRCM124333	CP116012.1
*Bacillus subtilis* strain MG‐1	CP110634.1
*Bacillus subtilis* BSn5	CP002468.1
*Bacillus subtilis* strain BIM B‐569G	CP069789.1
*Bacillus velezensis* strain AL7	CP045926.1
*Bacillus velezensis* strain ZeaDK315Endobac16	CP043809.1
*Bacillus* spp. *Pc3*	CP010406.1
*Bacillus velezensis* strain Q‐426	CP102351.1
*Bacillus amyloliquefaciens* strain W0101	CP090477.1

### Determination of minimum inhibitory concentration of CFS on Gram‐positive and Gram‐negative strains

The antimicrobial effect of CFS on planktonic cells of the test strains was evaluated using the broth microdilution method as per Haney et al. (2021). Briefly, an overnight culture of each test strain was incubated in TSBg. A 0.5 McFarland standard of the bacterial suspension was prepared using a densitometer and each test strain was diluted to 1 × 10^6^ CFU/mL. Sterile water (200 μL) was added to the outer wells of a 96‐well plate, with exception of row 12, which contained sterile growth medium (200 μL) as a positive control. Sterile broth (100 mL) was added to test wells. CFS (200 mL) was added to the initial well and two‐fold serially diluted in subsequent wells. Bacterial suspensions (100 mL) were then added to each CFS test well, resulting in a final bacterial concentration of 5 × 10^5^ CFU/mL at working concentrations of CFS ranging from 0.78% to 50%. CFS concentrations from the two producer strains were assessed against each test strain in triplicate. A growth control of test strains without CFS, at the same cellular concentration was included. All 96‐well plates were incubated at 37°C for 16–20 h and, post‐incubation, test strain growth was measured at 600 nm in a plate reader (Multiskan FC, Thermoscientific, Dublin, Ireland). The average absorbance for all sterility control wells and bacterial growth within the microtiter plate was calculated. The values were defined as 0% bacterial (SC_OD600_) and bacterial (GC_OD600_) growth. The percentage bacterial growth was determined using the following formula, where SC = sterile control and GC = growth control.

$$\%\text{Bacterial growth}=100\times \left({\text{Sample}}_{\mathrm{OD}600}-{\mathrm{SC}}_{\mathrm{OD}600}\right)/\left({\mathrm{GC}}_{\mathrm{OD}600}-{\mathrm{SC}}_{\mathrm{OD}600}\right).$$

### Minimal biofilm inhibitory concentration

The biofilm inhibition effect of the CFS was determined using the broth microdilution method as per Wiegand et al. (Wiegand et al., 2008) with modifications from Haney et al. (2021). Following incubation, bacterial suspensions were standardized to a concentration of 1 × 10^8^ CFU/mL using 0.5 McFarland standards and further diluted to 1 × 10^6^ CFU/mL. The CFSs were serial diluted to concentrations between 0.78 and 50% in sterile broth in a 96‐well plate. Bacterial suspensions (100 mL) were mixed with serially diluted CFS in the 96‐well plate. A growth control of test strains without CFS, at the same cellular concentration, was included; all 96‐well plates were incubated at 37°C for 16–20 h. To quantify biofilm biomass, following incubation, a crystal violet assay was performed. Plates were removed from the incubator and inverted over a waste container to discard contents. Wells were washed three times with phosphate buffered saline (PBS; 200 μL) and the formed biofilm was fixed by drying for 1 h at 60°C. Crystal violet (1%; 200 μL) was added to each well and incubated at room temperature for 10 min. Plates were inverted to remove crystal violet, washed three times with distilled water (200 μL) and dried at room temperature. Glacial acetic acid (30%; 200 μL) was added to the wells to solubilize the crystal violet bound to the biofilm. Plates were read at 595 nm wavelength on a plate reader (Multiskan FC, Thermoscientific, Dublin, Ireland). Values were defined as 0% bacterial growth (SC_OD600_), 100% biofilm growth (GC_A595_) or 0% biofilm growth (SC_A595_) during calculations. The percentage of bacterial growth and biofilm growth for each treatment was calculated using the following formulas (Haney et al., 2021):

$$\%\text{Bacterial growth}=100\times \left({\text{Sample}}_{\mathrm{OD}600}-{\mathrm{SC}}_{\mathrm{OD}600}\right)/\left({\mathrm{GC}}_{\mathrm{OD}600}-{\mathrm{SC}}_{\mathrm{OD}600}\right),$$

$$\%\text{Biofilm growth}=100\times \left({\text{Sample}}_{A595}-{\mathrm{SC}}_{A595}\right)/\left({\mathrm{GC}}_{A595}-{\mathrm{SC}}_{A595}\right).$$

Absorbance for biofilm wells was normalized against the negative control to determine if an MBIC_50_, MBIC_90_ or MBIC_99_, the lowest concentrations required to inhibit 50%, 90% and 99% of biofilm formation, respectively, had been achieved by each CFS concentration against each test strain. The average absorbance for all sterility wells within each microtiter plate was calculated for the average of crystal violet‐stained biomass (595 nm). Values were defined as 0% biofilm growth (SC_OD595_) and 100% biofilm growth (GC_A595_) during calculations. Formulae are as follows.

$${\text{MBIC}}_{99}=\left({\mathrm{GC}}_{A595}-{\mathrm{SC}}_{A595}\right)\times 0.01+{\mathrm{SC}}_{A595},$$

$${\text{MBIC}}_{90}=\left({\mathrm{GC}}_{A595}-{\mathrm{SC}}_{A595}\right)\times 0.1+{\mathrm{SC}}_{A595},$$

$${\text{MBIC}}_{50}=\left({\mathrm{GC}}_{A595}-{\mathrm{SC}}_{A595}\right)\times 0.5+{\mathrm{SC}}_{A59}.$$

### Minimum biofilm eradication concentration of CFS

To evaluate the effects of the CFS on established biofilms, a minimum biofilm eradication concentration (MBEC) assay was used Haney et al. (2021). Briefly, to allow biofilm formation to occur, 100 mL of bacterial suspension, adjusted to a concentration of 1 × 10^6^ CFU/mL, and 100 mL of sterile TSBg were added to each well of a microtiter plate, giving a final cell density of 5 × 10^5^ cells per well, and incubated overnight at 37°C. Following incubation, biofilm wells were washed three times using sterile PBS (200 mL) to remove planktonic cells. Fresh sterile TSBg (100 mL) and two‐fold serially diluted CFS (100 mL) was added to each mature biofilm well and plates were incubated for a further 24 h at 37°C. Bacterial suspensions (200 mL) were added to the negative control well at the same cell density as test wells. The bacterial growth for each well was recorded by measuring at 600 nm wavelength on the plate reader (Multiskan FC, Thermoscientific, Dublin, Ireland). Biofilm quantification was determined using the crystal violet assay as described above. The average absorbance for all sterility wells within each microtiter plate was calculated for both the bacterial growth (600 nm) and crystal violet‐stained biomass (595 nm) using the same calculations as for the MBIC and the thresholds for MBEC of 99, 90 and 50 were calculated in same manner as those for the MBIC calculations.

### Metabolic activity using the TTC reduction assay of CFS

The 2,3,5‐TTC‐triphenyl tetrazolium chloride (TTC) assay was performed to determine the metabolic activity of test strain biofilms as previously described by Haney et al. (2021) with some modifications. Briefly, biofilms were established in 96‐well microtiter plates as described above. Following incubation, planktonic cells were removed using sterile PBS. A two‐fold serially diluted CFS (100 mL) and sterile broth (100 mL) were added to each biofilm plate. TTC solution was prepared by dissolving TTC in distilled water to a final concentration of 0.05% (w/v) and filter sterilized using 0.2 μm pore size syringe filter. TTC solution (2 μL) was added to each well of the labelled TTC plate. All plates were incubated overnight at 37°C in the dark. Following incubation, the contents of the plates were discarded, and the plates were rinsed three times with PBS. Methanol (200 mL) was added to each well to dissolve the TTC dye. The plates were placed on an orbital shaker for ~30 min and the metabolic activity was recorded at 500 nm on a plate reader (Multiskan FC, Thermoscientific, Dublin, Ireland). Biofilm metabolism for each treatment was determined using the following formula, where SC = sterile control and GC = growth control (Haney et al., 2021):

$$\%\text{Biofilm metabolism}=100\times \left({\text{Sample}}_{A500}-{\mathrm{SC}}_{A500}\right)/\left({\mathrm{GC}}_{A500}-{\mathrm{SC}}_{A500}\right).$$

### Statistical analysis

Statistical analysis was performed using R (v 4.2.2; 2022.12.0). A normality test was utilized to evaluate if the data for each assay was normally distributed. A Levene's test of homogeneity was performed to assess variance within variables. For normal data which assumed equal variance, an ANOVA and Bonferroni post hoc test were performed. For normal data that did not assume equal variance, an ANOVA and Games‐Howell post hoc test were performed. For non‐normally distributed data, a Kruskal–Wallis test was conducted, followed by a Conover‐Iman post hoc test with a Bonferroni adjustment. Treatments were considered to differ significantly at *p* < 0.05. All results are presented as mean ± standard error of the mean (SEM) of four biological replicates (*n* = 3 technical repeats in each biological repeat).

## RESULTS

### Isolation of microorganisms from saltwater aquariums and subsequent overlay assay

Eleven distinct colonies were selected, given an identification code, and spotted onto TSA plates to perform a deferred antagonism assay to determine antimicrobial activity against the different bacterial test strains: *S. aureus* (MRSA, MTU48, MTU100, MTU300 and MTUCC), *A. baumannii* Ab003, *A. baumannii* Ab0013 and *E. coli* K‐12. Two colonies which displayed antimicrobial activity against Gram‐positive (*S. aureus*) and Gram‐negative (*E. coli*) test strains were isolated, characterized and identified through biochemical techniques. Following characterization, selected isolates were subjected to WGS to gain a comprehensive understanding of their genetic composition allowing for further investigation focusing on the potential biofilm formation inhibition and biofilm eradication activity.

### Prediction of secondary metabolite gene clusters

**FIGURE 1:**
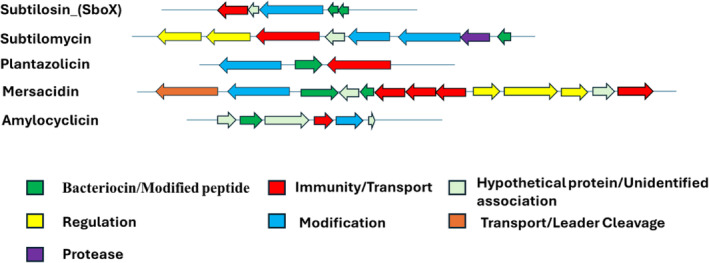
Diagrammatic representation of putative bacteriocin gene clusters of interest from *B. subtilis* MTUA2 (subtilosin_(SboX) and subtilomycin) and *B. velezensis* MTUC2 (plantazolicin, mersacidin and amylocyclin).

WGS identified two antimicrobial producers, *B. subtilis* and *B. velezensis*. Using the bacteriocin mining software tool, BAGEL 4, a total of nine areas of interest (AOIs) were initially identified from the two putative producer strains (Table 2). Based on WGS and genomic analysis, several bacteriocins were predicted to be produced by *B. subtilis* MTUA2 and *B. velezensis* MTUC2; however, the type of metabolites produced differed between the two strains. Subsequent manual annotation and protein BLAST (BLASTP) analysis determined that five of these were putative bacteriocin gene clusters (PBGCs) (Figure 1). Two PBGCs identified in *B. subtilis* MTUA2, subtilosin A and subtilomycin operons, encoded putative core peptides with 100% amino acid identity to that of the previously characterized subtilosin A and subtilomycin proteins (Phelan et al., 2013; Stein et al., 2004). Comparable analysis of *B. velezensis* MTUC2 identified three distinct PBGCs; plantazolicin, mersacidin and amylocyclicin operons, each showing similarities to previously characterized gene clusters (Altena et al., 2000; Scholz et al., 2014, 2011). Manual inspection of each operon found all key accessory genes required for synthesis were present. The remaining AOIs lacked core peptide or other key bacteriocin production‐associated genes.

**TABLE 2:** Identification of areas of interest (AOIs) by BAGEL 4 from *B. subtilis* MTUA2 and *B. velezensis* MTUC2 strains.

Strain	AOI	Nucleotide position		Type	Class	Identity
		From	To			
*Bacillus subtilis* MTUA2	CONTIG_6	174,134	198,629	Class 1 Lantipeptide	Subtilomycin^a^	100%
	CONTIG_2	375,176	395,789	Sactipeptide	Subtilosin_(SboX)^a^	100%
	CONTIG_4	227,273	247,273	Putative protein YfkA	Sactipeptides	100%
*Bacillus velezensis* MTUC2	CONTIG_9	27,344	51,781	RiPP (LAP)	Plantazolicin^a^	100%
	CONTIG_7	164,276	184,411	AMP	LCI	100%
	CONTIG_6	143,276	16,719	Lantibiotic	Mersacidin^a^	100%
	CONTIG_10	53,708	73,708	Putative protein YfkA	Sactipeptides	99.91%
	CONTIG_10	601,898	622,231	Bacteriocin	Amylocyclicin^a^	100%
	CONTIG_10	652,262	672,376	Competence pheromone	ComX4	100%

^a^ PBGCs with core peptide and all necessary accessory genes present.

Additional bioinformatic analysis was conducted using antiSMASH 7.0 to identify BGCs within the genomes of the isolates. Analysis showed that *B. subtilis* MTUA2 and *B. velezensis* MTUC2 possess nine and five gene clusters, respectively (Table 3). A comparison of known gene clusters in *B. subtilis* MTUA2, revealed six gene clusters that are involved in non‐ribosomal peptide synthetase (NRPS) activity, four trans‐acyl transferase polyketide synthetase (transAT‐PKS), two terpene, two type III polyketide synthetases (T3PKS), one lantipeptide class‐1 and one sactipeptide. *Bacillus velezensis* MTUC2 encoded six clusters involved in NRPS, five transAT‐PKS, two terpene, and one T3PKS. Another notable distinction between the two strains were the presence of the lantipeptide class 1 and the sactipeptides responsible for the synthesis of subtilosin and subtilomycin; these specific peptides were absent in *B. velezensis* MTUC2. The lantipeptide class II and the LAP (linear azol(in)e‐containing peptides) responsible for synthesizing mersacidin and plantazolicin in *B. velezensis* were not present in the *B. subtilis* MTUA2 isolate.

**TABLE 3:** Comparative analysis with antiSMASH 7.0 of secondary metabolite gene clusters of *B. subtilis* MTUA2 with *B. velezensis* MTUC2.

Type	Most similar known cluster	Biosynthetic class(es)	*Bacillus subtilis* MTUA2% identity	*Bacillus velezensis* MTUC2% identity
terpene	‐	‐	‐	‐
transAT‐PKS PKS‐like T3PKS NRPS	Bacillaene	Polyketide+NRP	100%	100%
Sactipeptide	Subtilosin A	RiPP Thiopeptide	100%	‐
T3PKS	1‐carbapen‐2‐em‐3‐carboxylic acid	other	16%	‐
LAP	Plantazolicin	RiPP Lantipeptide	‐	91%
NRPS betalactone	Fengycin	NRP	100%	86%
NRPS‐like	K53 capsular polysaccharide	Saccharide	10%	‐
NRPS	Surfactin	NRP‐lipopeptide	56%	91%
lantipeptide‐class‐1	Subtilomycin	RiPP Lantipeptide	100%	‐
PKS‐like	butirosin A/butirosin B	Saccharide	‐	7%
transAT‐PKS	Macrolactin	Polyketide	‐	100%
lantipeptide‐class‐1	Mersacidin	RiPP Lantipeptide	‐	100%
transAT‐PKS	Difficidin	Polyketide	‐	100%
NRPS transAT‐PKS	Bacinapeptin	RiPP	‐	50%
other	Bacilysin	other	100%	100%
NRP‐metallophore NRPS	Bacilibactin	NRP	100%	100%
NRPS	Pipastatin	NRP	‐	46%

*Note*: (‐) No reference values.

### *Minimum inhibitory concentrations of* B. subtilis *MTUA2 and* B. velezensis *MTUC2 CFS on planktonic cell growth of Gram‐positive and Gram‐negative pathogens*

**FIGURE 2:**
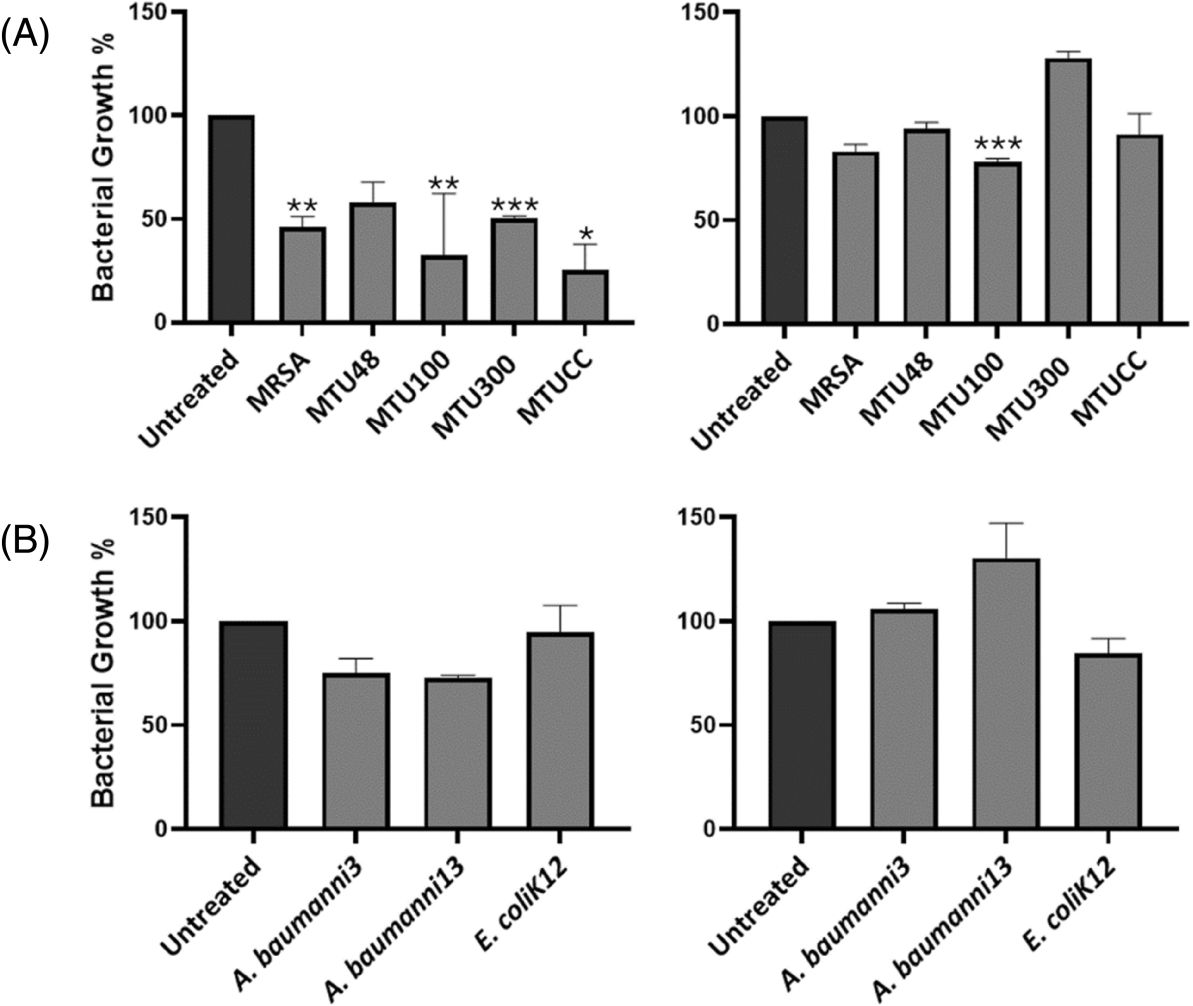
Antimicrobial activity of the cell free supernatant (CFS; 50% concentration) of *B. subtilis* MTUA2 (left) and *B. velezensis* MTUC2 (right) against (A) Gram‐positive and (B) Gram‐negative test strains was measured at A_600_. All experiments were performed in triplicate and the results are expressed as mean ± standard error (SEM). Significant difference is denoted at ***p < 0.001, **p < 0.01, *p < 0.05, compared to the untreated control (denoted as 100% bacterial growth).

The effect of *B. subtilis* MTUA2 and *B. velezensis* MTUC2 CFS against planktonic cell growth was assessed against Gram‐positive MRSA and *S. aureus* clinical isolates (MTU48, MTU100, MTU300, MTUCC) and Gram‐negative *E. coli* K‐12, *A. baumannii* Ab003 and *A. baumannii* Ab0013 strains (Figure 2). The results showed that in the presence of a 50% concentration of *B. subtilis* MTUA2 CFS, the planktonic cell growth of all Gram‐positive test strains was inhibited with significant inhibition observed for the clinical isolates, MTU300 (*p* < 0.001), MRSA, MTU100 (*p* < 0.01) and MTUCC (*p* < 0.05), compared to the untreated control. *Bacillus velezensis* MTUC2 CFS was less effective, reducing planktonic cell growth of only the clinical isolate MTU100 (*p* < 0.001) and was observed to increase planktonic cell growth of the clinical isolate MTU300. A marginal effect was observed for 50% *B. subtilis* MTUA2 CFS against the planktonic cell growth of *E. coli* and *A. baumannii* test strains; however, *B. velezensis* MTUC2 did not demonstrate any notable antimicrobial activity against these test strains.

### Minimum biofilm inhibitory concentration of CFS on Gram‐positive and Gram‐negative test strains

To investigate the potential of *B. subtilis* MTUA2 and *B. velezensis* MTUC2 CFS to inhibit biofilm formation, biofilm mass was quantitatively determined following 24 h incubation with CFS treatment. Subsequent MBIC_50_, MBIC_90_ and MBIC_99_ values are presented in Table 4. The CFS of both strains successfully prevented biofilm formation with an MBIC_50_ achieved for all tested Gram‐positive strains. *Bacillus subtilis* MTUA2 significantly inhibited biofilm formation of the *S. aureus* clinical test strains MTU48, MTUCC (*p* < 0.001), MTU100 and MRSA (*p* < 0.01), achieving a mbic_99_ of the *S. aureus* clinical isolate MTU48 with the 50% CFS concentration. *Bacillus subtills* MTUA2 demonstrated superior efficacy in inhibiting the formation of biofilm by the clinical isolate MTU300 achieving an MBIC90 with 12.5% CFS, compared to 25% required for *B. velezensis* MTUC2. Interestingly, *B. velezensis* MTUC2 demonstrated a greater ability to prevent biofilm formation against MRSA and MTUCC achieving an MBIC_90_ with 6.25 and 50% CFS concentrations, respectively.

**TABLE 4:** Minimum biofilm inhibitory concentration values of saltwater isolates against all test strains.

Microorganisms	*Bacillus subtilis* MTUA2			*Bacillus velezensis* MTUC2		
	CFS conc. % (v/v)					
	MBIC_99_	MBIC_90_	MBIC_50_	MBIC_99_	MBIC_90_	MBIC_50_
*Staphylococcus aureus* MTU48	50	50	3.125	‐	50	0.78
*Staphylococcus aureus* MTU100	‐	‐	0.78	‐	NA	12.5
*Staphylococcus aureus* MTU300	‐	12.5	12.5	‐	25	25
*Staphylococcus aureus* MTUCC	‐	25	6.25	‐	6.25	3.125
MRSA	‐	‐	1.56	‐	50	1.56
*Acinetobacter baumannii* Ab003	‐	‐	25	‐	‐	‐
*Acinetobacter baumannii* Ab0013	‐	‐	12.5	‐	‐	0.78
*Escherichia coli* K‐12	‐	‐	12.5	‐	25	6.25

*Note*: Values are the means of four biological repeats (*n* = 3 technical repeats in each biological repeat). (‐) MBIC not achieved.

Both *B. subtilis* MTUA2 and *B. velezensis* MTUC2 demonstrated the ability to inhibit biofilm formation of the Gram‐negative bacteria tested. *Bacillus subtilis* MTUA2 significantly (*p* < 0.001) reduced biofilm formation by *A. baumannii* Ab003, compared to the untreated control, achieving an MBIC_50_ with 25% CFS concentration. In addition, *B. subtilis* MTUA2 inhibited biofilm formation by *E. coli* K‐12 and *A. baumannii* Ab0013, approaching statistical significance at MBIC_50_. *Bacillus velezensis* MTUC2 significantly prevented biofilm formation of Gram‐negative bacteria with the most significant effect observed against *E. coli* K‐12, achieving an MBIC_50_ at a lower CFS concentration of 6.25%. *Bacillus velezensis* MTUC2 demonstrated inhibition of *A. baumannii* Ab0013 biofilm formation MBIC_50_; however, this was not deemed statistically significant.

### *Effect of* B. subtilis *MTUA2 and* B. velezensis *MTUC2 CFS on established bacterial biofilm*

**FIGURE 3:**
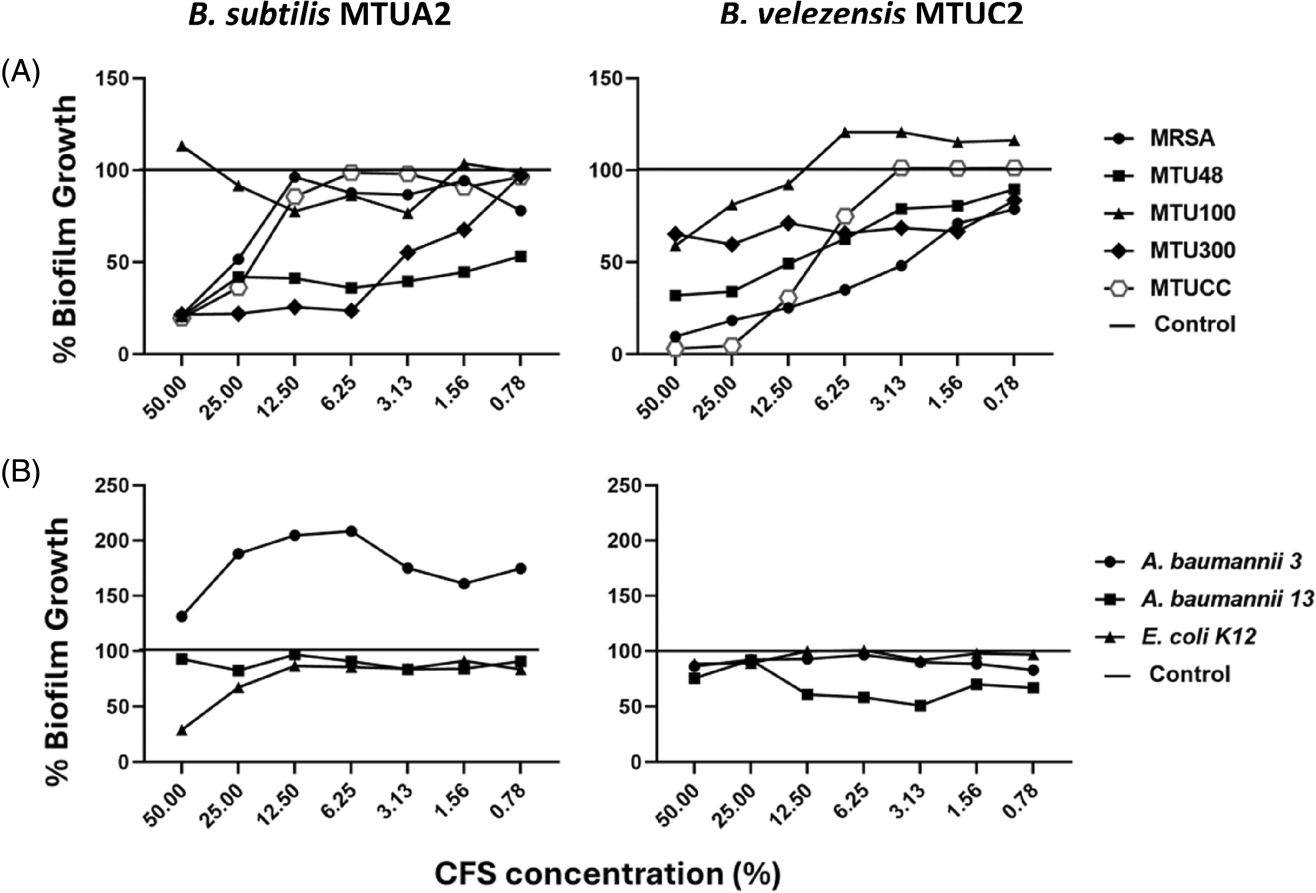
Erradication effect of *B. subtilis* MTUA2 (left) and *B. velezensis* MTUC2 (right) CFS on pre‐formed biofilms of (A) Gram‐positive and (B) Gram‐negative test strains. Following exposure to CFS, the pre‐formed biofilms were incubated at 37°C for 24 h. The pre‐formed biofilms of both Gram‐positive and Gram‐negative test strains were stained with 1% crystal violet and assessed by measuring at A_595_. Results are expressed as mean compared to the untreated control denoted as 100% bacterial growth. Values are the means of four biological repeats (*n* = 3 technical repeats in each biological repeats).

As bacterial biofilms are considered the main cause of chronic infections (Bjarnsholt, 2013; Ilyina & Romanova, 2021), the ability of *B. subtilis* MTUA2 and *B. velezensis* MTUC2 to eradicate pre‐formed biofilms was investigated and the results are presented in Figure 3. All Gram‐positive test strains, except clinical isolate MTU100, demonstrated reduced biofilm mass when treated with *B. subtilis* MTUA2 CFS. This reduction was significant (*p* < 0.001) for the biofilm biomass of MTU300 and MTUCC clinical isolates. Similar results were observed for *B. velezensis* MTUC2; however, there was a notable difference against MRSA and the clinical isolate MTUCC with a significant (*p* < 0.001) reduction in biomass. *Bacillus velezensis* MTUC2 also appeared effective in a dose dependent manner. In the presence of 25% *B. velezensis* MTUC2 CFS, the mature biofilm of MTUCC was reduced to almost undetectable levels compared to 50% needed by *B. subtilis* MTUA2. *Bacillus velezensis* MTUC2 was also shown to be more effective at significantly reducing MTU48 and MRSA biofilms at 25% and 50%, respectively, while no significant biofilm reduction was observed for *B*. *subtilis* MTUA2. Contrastingly, compared to *B. velezensis* MTUC2, *B. subtilis* MTUA2 reduced MTU300 biofilm biomass at the lower concentration of 6.25% CFS.

Against established Gram‐negative test strains, *B. subtilis* MTUA2 significantly (*p* < 0.01) reduced *E. coli* K‐12 biofilm mass by ~70% at 50% CFS concentration, whereas *B. velezensis* MTUC2 had no effect. In contrast, *B. subtilis* MTUA2 had no effect on *A. baumannii* isolates, whereas *B. velezensis* MTUC2 marginally reduced *A. baumannii* Ab0013 biomass at 3.13% CFS concentration. Interestingly, at higher concentrations *A. baumannii* Ab0013 remained unaffected. Both *B. subtilis* MTUA2 and *B. velezensis* MTUC2 demonstrated no antibiofilm effect against *A. baumannii* Ab003; in fact, when treated with *B. subtilis* MTUA2 there was a notable increase in biofilm biomass in comparison to the untreated control.

### Effect of CFS on metabolic activity on Gram‐positive and Gram‐negative pathogens

**FIGURE 4:**
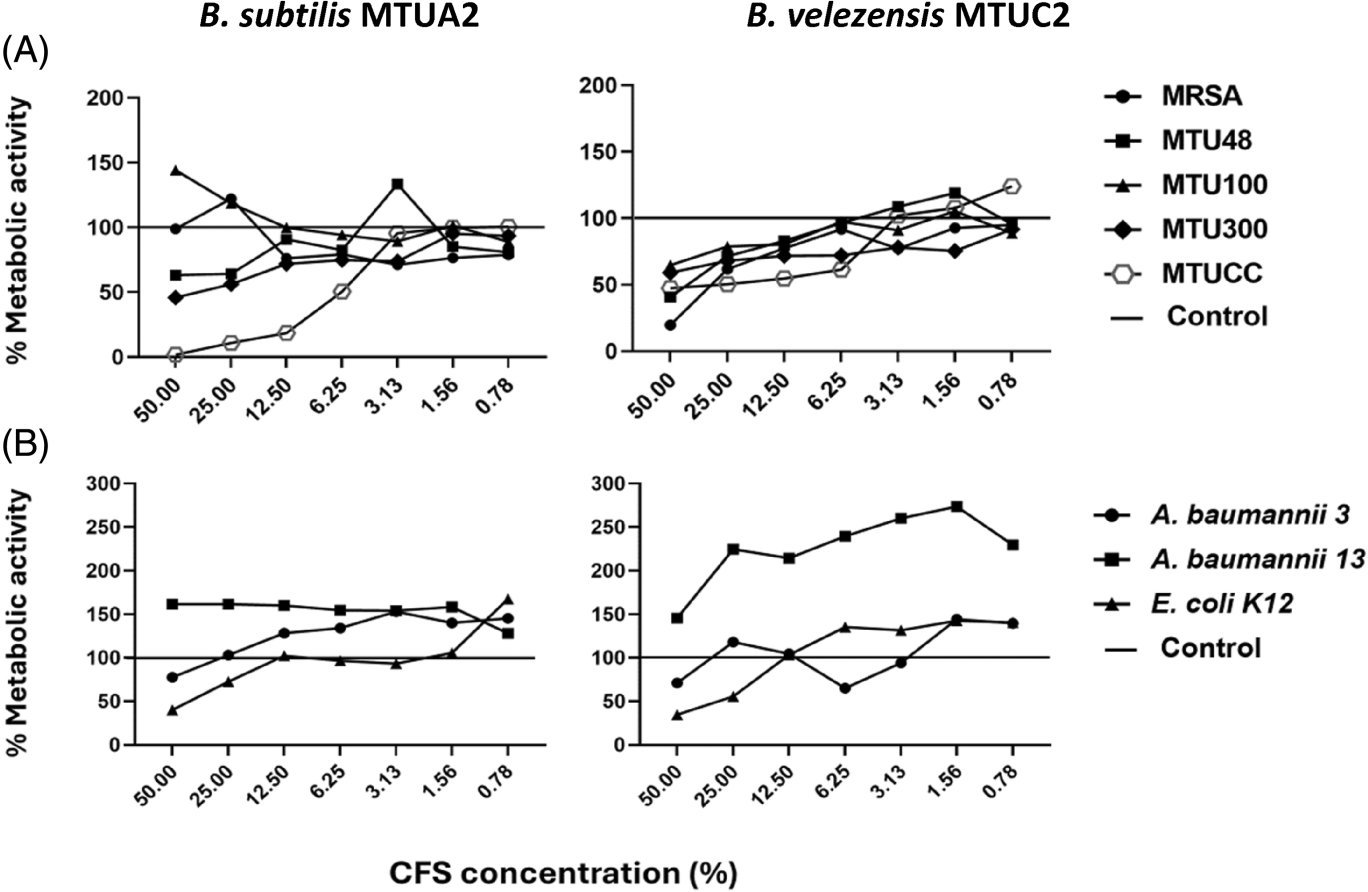
Effect of *B. subtilis* MTUA2 and *B. velezensis* MTUC2 CFS on the metabolic activity of (A) Gram‐positive and (B) Gram‐negative biofilm cells. The established biofilms of Gram‐positive and Gram‐negative test strains were incubated in the presence of CFS concentrations at 37°C for 24 h. The metabolic activity of Gram‐positive and Gram‐negative biofilm cells were a‐lysed using the TTC reduction assay and assessed by measuring as A_500_. The results are expressed as mean compared to the untreated control denoted as 100% bacterial growth. Values are the means of four biological repeats (*n* = 3 technical repeats in each biological repeat).

A TTC assay was used to approximate the metabolic activity remaining within the biofilm when treated with *B. subtilis* MTUA2 and *B. velezensis* MTUC2 CFS. Treatment with 50% *B. subtilis* MTUA2 CFS resulted in a notable decrease in the metabolic activity of three *S. aureus* clinical isolates. The metabolic activity of clinical isolate MTUCC exhibited almost complete reduction, with a 99% decrease (*p* < 0.001) (Figure 4A). Results show that *B. subtilis* MTUA2 reduced the metabolic activity of the test strain *E. coli* K‐12 by <50% with 50% CFS. In addition, a notable reduction was observed in the metabolic activity of the *A. baumannii* Ab003 test strain. In contrast, *B. velezensis* MTUC2 CFS reduced the metabolic activity of all Gram‐positive test strains in a more dose dependent manner (Figure 4A). The metabolic activity of the clinical isolate MTUCC (*p* < 0.001), MRSA and the clinical isolate MTUCC (*p* < 0.01) were significantly reduced with 50% CFS, compared to the untreated control. While there was no statistically significant reduction in *A. baumannii* metabolic activity when treated with *B. velezensis* MTUC2, it significantly reduced (*p* < 0.01) metabolic activity of *E. coli* K‐12 within an established biofilm with 25% CFS concentration (Figure 4B).

## DISCUSSION

The rapid increase in antibiotic resistance has intensified the upsurge in hospital‐acquired infections in recent years contributing to the increased transmission of these infections within healthcare settings. Pathogens causing these infections are frequently classified by their capacity to develop biofilms which exhibit features different from those of free‐living planktonic cells, such as a different physiology and enhanced antibiotic resistance (Lewis, 2007; Mulani et al., 2019; Tong et al., 2015). Subsequently, biofilms have become a source of persistent and chronic infections (Talebi Bezmin Abadi et al., 2019). The primary objective of this study was to identify bacteria with the capacity to inhibit growth and biofilm formation of a range of Gram‐positive and Gram‐negative ESKAPE pathogens, broadly associated with antibiotic resistance. This study identified two *Bacillus* species, *B. subtilis* MTUA2 and *B. velezensis* MTUC2, isolated from saltwater aquariums with antimicrobial activity. It has been widely documented that bacteria in the genus *Bacillus* have the potential to simultaneously produce an array of substances with antimicrobial and antibiofilm properties (Afroj et al., 2021; Tareq & Shin, 2017). Over the range evaluated, this study showed that the CFS of both strains reduced bacterial growth and inhibited biofilm formation of both Gram‐positive and Gram‐negative test strains (Figure 2 and Table 4). Additionally, substantial antibiofilm activity was observed against established biofilms, as evidenced by a reduction in both biofilm mass and metabolic activity within the biofilm (Figures 3 and 4). However, a difference in effectiveness was observed between these two *Bacillus* strains, likely attributable to variations in the secondary metabolites they produced. *Bacillus subtilis* MTUA2 was predicted to produce two key bacteriocins, subtilomycin and subtilosin A, while *B. velezensis* MTUC2 was predicted to produce a different suite of bacteriocins, including, plantazolicin, mersacidin and amylocyclicin along with the signalling peptide ComX4.

Consistent with previous studies (Lotfy et al., 2018; Ramachandran et al., 2014; Sharma et al., 2019), this study found that *B. subtilis* MTUA2 exerted broad spectrum antimicrobial activity, against Gram‐positive MRSA and *S. aureus* and Gram‐negative *E. coli* and *A. baumannii*; however, the latter showed more modest susceptibility. *Bacillus velezensis* MTUC2 showed antimicrobial activity against some Gram‐positive test strains with a significant antimicrobial effect against the *S. aureus* clinical isolate MTU100 with no notable antimicrobial action against the Gram‐negative test strains assayed (Figure 2). From genome sequencing (Tables 3 and 4), *B. subtilis* MTUA2 was predicted to produce two key PBGCs, subtilosin A and subtilomycin, previously shown to play a key role in targeting planktonic bacteria (Babasaki et al., 1985; Phelan et al., 2013; Turovskiy et al., 2012). While the antimicrobial effect of *B. velezensis* MTUC2 might also be attributed to the potency of the different suite of bacteriocins synthesized. Notably, Paradies et al. (2019) demonstrated the ultra‐narrow spectrum activity of plantazolicin. Plantazolicin exerts its action by disrupting the integrity of the cell membrane with potent activity, especially towards *B. anthracis* (Molohon et al., 2016), while the lantibiotic amylocyclicin exhibited robust antagonistic potential against predominantly Gram‐positive pathogens (Müller et al., 2016). Lantibiotics, in general, target Gram‐positive bacteria, which typically involve binding to the cells wall precursor lipid ll and disrupting cell wall synthesis (Wiedemann et al., 2001). However, there are instances where lantibiotics have demonstrated some antimicrobial activity against Gram‐negative bacteria, such as *E. coli*, *Moraxella catarrhalis* and *Neisseria* spp. (Castiglione et al., 2008; Draper et al., 2015). The antibiofilm effect of both strains was comparable, achieving an MBIC_50_ for all Gram‐positive and Gram‐negative pathogens with the exception of *B. velezensis* against *A. baumannii* Ab003 (Table 4). Interestingly, *B. velezensis* MTUC2 inhibited the biofilm formation of *A. baumannii* Ab0013 *and E. coli* K‐12 at lower CFS concentrations (0.78% and 6.25%), respectively, compared to *B. subtilis* MTUA2. These results, in line with previous studies, suggest that the antibiofilm effects of both strains may be attributed to the presence of three gene clusters involved in non‐ribosomal synthesis of bacillaene, bacilysin and bacilibactin; these clusters have previously been shown to contribute to biofilm inhibition (Dimopoulou et al., 2021; Erega et al., 2021). Compared to *B. velezensis* MTUC2, *B. subtills* MTUA2 displayed stronger activity against *A. baumannii* achieving an MBIC_50_ for both test strains. Genomic analysis predicted the potential production of two distinct metabolites by *B. subtilis* MTUA2, 1‐carbapen‐2‐em‐3‐carboxylic acid, a known broad‐spectrum antibiotic and K53 capsular polysaccharide (Table 3). Conversely, genome sequencing results revealed the production of ComX by *B. velezensis*. ComX can prevent QS by disrupting cell signalling suggesting its potential contribution to the antibiofilm effect. Previously, it has been shown that ComX4, a key component of a QS system that controls the development of genetic competence (Okada et al., 2005) and the synthesis of surfactin (Caulier et al., 2019), inhibited biofilm formation of *S. aureus* (Liu et al., 2019). These results suggest that biofilm inhibition by both *B. subtilis* MTUA2 and *B. velezensis* MTUC2 may be due to different metabolites produced by each strain. Further to this, we investigated the ability of *B. subtilis* MTUA2 and *B. velezensis* MTUC2 to reduce preformed biofilms. A reduction of biofilm biomass was observed against all Gram‐positive established biofilms, with the exception of *B. subtilis* MTUA2 against the biofilm of *S. aureus* clinical isolate MTU100 (Figure 4). *Bacillus subtilis* MTUA2 was observed to disrupt some Gram‐negative test strains, marginally reducing *A. baumannii* Ab0013, with a notable reduction in *E. coli* K‐12 biofilms by ~70%. *Bacillus subtilis* has been shown to exert its antibiofilm activity against Gram‐negative test strains such as *Gardnerella vaginalis* by producing the lantibiotic subtilosin, which has been found to reduce the production of autoinducers and QS molecules (Algburi et al., 2017). *Bacillus velezensis* MTUC2 proved to be more comprehensive against established Gram‐positive established biofilms with near total removal of MRSA and MTUCC biofilms. This may be a result of a higher surfactin content, a type of biosurfactant produced by *Bacillus*, previously shown to disrupt established biofilm (Allegrone et al., 2021). However, there was no significant disruptive effect evident against the Gram‐negative test strains used. As previously mentioned, we investigated the effect of *B. subtilis* MTUA2 and *B. velezensis* MTUC2 CFS on the metabolic rate of Gram‐positive and Gram‐negative test strains (Figure 4). Certain cells living in biofilms can transform into specialized cells called persister cells which are metabolically dormant and resistant to antibiotic treatments. Unlike unhealthy or dying cells, persister cells maintain structural integrity and can resume growth when conditions are favourable (Bartell et al., 2020; Lewis, 2007; Wainwright et al., 2021). This study revealed that *B. subtilis* MTUA2 reduced the metabolic rate in Gram‐positive and Gram‐negative bacterial test strains, with a statistically significant reduction in the metabolic activity of the *S. aureus* clinical isolate MTUCC and the Gram‐negative test strain *E. coli* K‐12. The production of secondary metabolites may have been responsible for the metabolic decline rate. For instance, fengycins, cyclic lipopeptides produced by *Bacillus* species have been shown to exert strong antibacterial activity against a range of pathogenic bacteria by damaging the cellular envelope may have contributed to this metabolic decline. Fengycins cause leakage of intracellular content and damage to the cell membrane and cell wall, ultimately leading to cell death (Medeot et al., 2020). However, in this study, the metabolic activity of the Gram‐positive *S. aureus* MRSA, and clinical isolates MTU100 and MTUCC appeared to increase in the presence of high concentrations of *B. subtilis* MTUA2 CFS. This may be a result of a subset of cells within the biofilm adopting persister cell physiology in response to antimicrobial stress (Bartell et al., 2020; Choueiry et al., 2022). This transition could demand additional energy and resources, temporarily increasing the metabolic activity as cells prepare to resume growth and respond to environmental changes (Wainwright et al., 2021). However, the same response was not observed for *B. velezensis* MTUC2 which reduced the metabolic rate of cells within established biofilms of all Gram‐positive test strains with a statistically significant reduction against MRSA and the *S. aureus* clinical isolates MTU48 and MTUCC. This could be a consequence of the presence of macrolactin, a class of antibiotic synthesized by *B. velezensis*, known to possess strong antibacterial properties (Yuan et al., 2016) against many bacterial pathogens. Interestingly, compared to *B. subtilis* MTUA2, *B. velezensis* MTUC2 did reduce the metabolic activity of the Gram‐negative *E. coli* K‐12 test strain, whereas the metabolic rates of the *A. baumannii* test strains were more erratic over the CFS concentration range; cells appeared to become metabolically less inactive with 6.75% CFS concentrations but regained a substantial level of metabolism at higher concentrations (25%). This would again suggest the resilient nature of *A. baumannii* species to adapt to antimicrobial agents (Harding et al., 2018; Perez et al., 2007). Studies have found that *A. baumannii* can form biofilms at a rate up to three times faster than other *Acinetobacter* species (Sung, 2018). The variance between the two producer strains to reduce metabolic activity of the test strains may be a consequence of interactions between the unique metabolites produced by both strains investigated. This interaction is particularly relevant in the context of new research on the therapeutic potential of *B. subtilis* and *B. velezensis*. For instance, a recent study investigating the therapeutic potential of the combined action of *B. subtilis* CFS and polymyxin E have shown a synergistic effect, demonstrating significant effectiveness in inhibiting the formation of *Acinetobacter* spp. biofilms (AL‐Dulaimi et al., 2021). Another study by Suchi et al. (2023) demonstrated the ability of an antimicrobial peptide, YS12, isolated from *B. velezensis* CBSYS12 which exerts antibiofilm properties against multidrug resistant Gram‐positive and Gram‐negative bacteria such as *E. coli*, *P. aeruginosa*, MRSA, vancomycin‐resistant *Enterococcus* (VRE) and *Mycobacterium smegmatis*. This peptide exhibits superior biofilm eradication activity compared to commercial antibiotics (Dimopoulou et al., 2021.). These examples reflect the extensive and continuous exploration of *Bacillus* CFS in the context of potential therapeutic agents, with significant implications for addressing infections associated with biofilms.

In conclusion, our findings demonstrate that CFSs derived from *B. subtilis* MTUA2 and *B. velezensis* MTUC2 exert antimicrobial and antibiofilm effects on both Gram‐positive and Gram‐negative ESKAPE pathogens. These effects appear to be related to the specific secondary metabolites produced by each strain. This study reinforced the value of *B. subtilis* and *B. velezensis* broad‐spectrum antimicrobials and suggests the potential of *B. subtilis* and *B. velezensis* as new therapeutic alternatives against a range ESKAPE pathogens. Nevertheless, additional studies are essential to determine the mechanisms through which *B. subtilis* and *B. velezensis* prevent the formation of, or disrupt, established biofilms, with further investigations warranted to elucidate their combined or synergistic effectiveness when used in conjunction with conventional antibiotics. Such studies could expand insight into developing new antimicrobial and antibiofilm agents to treat biofilm‐associated infections caused by multidrug resistant ESKAPE pathogens.

## AUTHOR CONTRIBUTIONS

**Monica M. Murphy:** Investigation; methodology; conceptualization; writing – original draft; visualization; data curation; formal analysis. **Eamonn P. Culligan:** Writing – review and editing; funding acquisition; supervision. **Craig P. Murphy:** Funding acquisition; writing – review and editing; supervision.

## CONFLICT OF INTEREST STATEMENT

The authors declare no conflicts of interest.

## Data Availability

Data available on request.
